# Interferon gamma-induced apoptosis of head and neck squamous cell carcinoma is connected to indoleamine-2,3-dioxygenase *via* mitochondrial and ER stress-associated pathways

**DOI:** 10.1186/s13008-016-0023-4

**Published:** 2016-08-02

**Authors:** Siraj M. El Jamal, Erin B. Taylor, Zakaria Y. Abd Elmageed, Abdulhadi A. Alamodi, Denis Selimovic, Abdulaziz Alkhateeb, Matthias Hannig, Sofie Y. Hassan, Simeon Santourlidis, Paul L. Friedlander, Youssef Haikel, Srinivasan Vijaykumar, Emad Kandil, Mohamed Hassan

**Affiliations:** 1 Department of Pathology, University of Mississippi Medical Center, Jackson, MS 39216 USA; 2 Department of Physiology and Biophysics, University of Mississippi Medical Center, Jackson, MS 39216 USA; 3 Departments of Surgery, Tulane University School of Medicine, New Orleans, LA 70112 USA; 4 Clinic of Operative Dentistry, Periodontology and Preventive Dentistry, Saarland University, Kirrberger Str. 100, 66421 Homburg/Saar, Germany; 5 Division of Oral Health Science, Department of Restorative Dentistry, Graduate School of Dental Medicine, Hokkaido University, Sapporo, Japan; 6 Clinic of Dermatology, University Hospital of Aachen, Puwelstrasse 30, Aachen, Germany; 7 College of Medicine, King Faisal University, Alhofuf, Saudi Arabia; 8 Epigenetics Core Laboratory, Institute of Transplantation Diagnostics and Cell Therapeutics, University Hospital of Duesseldorf, Heinrich-Heine-University of Duesseldorf, Mooren Str.5, 40225 Duesseldorf, Germany; 9 Institut National de la Santé et de la Recherche Médicale, University of Strasbourg, 67000 Strasbourg, France; 10 Department of Operative Dentistry and Endodontics, Dental Faculty, University of Strasbourg, 67000 Strasbourg, France; 11 Department of Radiation Oncology, University of Mississippi Medical Center, Jackson, MS 39216 USA; 12 Cancer Institute, University of Mississippi Medical Center, Jackson, MS 39216 USA

**Keywords:** HNSCC, IDO, HO-1, FN-γ, JAK, STAT1

## Abstract

**Background:**

Tumor response to immunotherapy is the consequence of a concerted crosstalk between cytokines and effector cells. Interferon gamma (IFNγ) is one of the common cytokines coordinating tumor immune response and the associated biological consequences. Although the role of IFNγ in the modulation of tumor immunity has been widely documented, the mechanisms regulating IFNγ-induced cell death, during the course of immune therapy, is not described in detail.

**Results:**

IFNγ triggered apoptosis of CLS-354 and RPMI 2650 cells, enhanced the protein expression and activation of indoleamine 2,3-dioxygenase (IDO), and suppressed the basal expression of heme oxygenase-1(HO-1). Interestingly, IFNγ induced the loss of mitochondrial membrane potential (Δ*ψ*m) and increased accumulation of reactive oxygen species (ROS). The cytokine also induced the activation of Janus kinase (JAK)/Signal Transducer and Activator of Transcription (STAT)1, apoptosis signal-regulating kinase 1 (ASK1), p38, c-jun-N-terminal kinase (JNK) and NF-κB pathways and the transcription factors STAT1, interferon regulatory factor 1 (IRF1), AP-1, ATF-2, NF-κB and p53, and expression of Noxa protein. Furthermore, IFNγ was found to trigger endoplasmic reticulum (ER) stress as evidenced by the cleavage of caspase-4 and activation of protein kinase RNA-like endoplasmic reticulum kinase (PERK) and inositol-requiring-1α (IRE1α) pathways. Using specific inhibitors, we identified a potential role for IDO as apoptotic mediator in the regulation of IFNγ-induced apoptosis of head and neck squamous cell carcinoma (HNSCC) cells via Noxa-mediated mitochondrial dysregulation and ER stress.

**Conclusion:**

In addition to the elucidation of the role of IDO in the modulation of apoptosis, our study provides new insights into the molecular mechanisms of IFNγ-induced apoptosis of HNSCC cells during the course of immune therapy.

## Background

The tumor response to immunotherapy is the consequence of a concerted crosstalk between cytokines and effector cells [1]. Interferon gamma (IFNγ) is one of the central cytokines that coordinates tumor immune responses and the associated biological consequences [2]. IFN-γ is a pleiotropic cytokine with multiple biological functions including immune cell activation [3, 4] and induction of the major histocompatibility complex (MHC) molecules both in normal and neoplastic cells [5, 6]. Besides its ability to trigger cell cycle arrest and apoptosis [7–10], IFN-γ shows anti-tumor activity in patients with advanced head and neck squamous cell carcinoma (HNSCC) [11] or non-small-cell lung carcinoma (NSCLC) [12]. Although there is clinical data highlighting the reliability of IFN-γ as an anti-tumor agent [10], the molecular action of IFN-γ as anti-cancer agent has not been fully investigated. Because the current reported studies on the molecular action of IFN-γ in tumor cells are merely speculative, the aim of the present study is to elucidate, in detail, the mechanisms which are responsible for the modulation of IFN-γ-induced effects on HNSCC cells.

IFNγ coordinates cellular functions via transcriptional regulation of innate and adaptive immune response-associated genes such as indoleamine 2,3-dioxygenase (IDO) [13]. IDO is a rate limiting enzyme in the kynurenine enzymatic pathway that converts tryptophan (Trp) to *N*-formyl-kynurenine, which is the main source for the production of the cellular cofactor NAD^+^ [14]. While IDO has been found to be constitutively expressed in a limited number of human tissues [15], its induction and activation are tissue-specific and agent-dependent [15–17]. The induction of IDO by lipopolysaccharides (LPS), IFNγ, tumor necrosis factor alpha (TNF-α) and Fas receptor agonist (CH11) has been reported in different cell types [15, 18–20]. An in vivo induction of IDO is associated with IFNγ-mediated inflammation that mediates the innate immune response to intracellular pathogens and bacterial infection [21–24]. IDO is expressed in a wide array of human cancers [25], and the contribution of IDO in the regulation of tumor cell death has been demonstrated in several studies [18, 19, 26, 27]. In accordance, it is expected that IDO contributes to the regulation of IFNγ-induced cell death.

Herein we demonstrate an essential role for IDO as an apoptotic mediator during the course of IFNγ-induced death of HNSCC cells via a mechanism mediated by IDO-dependent suppression of HO-1.

## Methods

### Assessment of cell survival

Human head and neck squamous cell carcinoma (HNSCC), CLS-354 CLS (Cell Lines Service GmbH, Germany) and SCC nasal septum, RPMI 2650 (ATCC^®^ CCL-30™) obtained from the American Tissue Culture Collection (ATCC, Manassas, VA, USA) were seeded in 96-microwell plates (1 × 10^4^ cells/well), (Nunc, Waltham, MA, USA). The cells were challenged with IFNγ (1000 U/ml) for the indicated time periods. The percentage of viable cells was then determined using the colorimetric MTT assay (Roche, Bâle, Switzerland) as described [28].

### RNA interference

The knockdown of IDO gene was performed using siRNA, and negative control siRNA as described in the manufacturer’s protocol (Qiagen, Hilden; Germany). The transfection of the cell lines was performed using lipofectamine 2000 (Invetrogen) as described [29].

### Assay for intracellular IDO activity

The CLS-354 and RPMI 2650 cell lines (1 × 10^4^) were allowed to grow overnight in a 24-well plate. The cells were treated with IFNγ (1000 U/ml) for 48 h. The treated and control cells were harvested and washed three times in phosphate buffered saline (PBS). The cell pellet was snap frozen at −70 °C and next day was homogenized in 0.5 ml PBS. Centrifugation was performed at 15,000×*g* for 10 min at 4 °C, then the supernatant was carefully collected for measuring IDO activity as previously described [18]. Briefly, the cell extract was mixed with 100 μl reaction buffer (100 mM potassium phosphate buffer pH 6.5, 40 mM ascorbate, 20 μM methylene blue, 200 μg/ml catalase, 800 μM l-tryptophan). This step followed by incubation at 37 °C to activate the IDO enzyme to convert l-tryptophan to *N*-formyl-kynurenine. About 30 min later, the termination of the reaction was performed following the addition of 49 μl of trichloracetic acid (30 %). Following hydrolysis process of *N*-formyl-kynurenine to kynurenine has been completed, Then the measurement of the enzyme activity was performed by mixing of 100 μl of reaction mixture together with 100 μl Ehrlich reagent (0.4 % *p*-methyaminobenzaldehyde/acetic acid) using a Microplate reader (ImmunoReader NJ-2000 Nunc, Wiesbaden, Germany). The absorbance was at 490 nm.

### Immunoblot

Protein analysis was performed using standard immune blotting. The following antibodies were used at the indicated dilution: anti-Noxa (SC-2697) 1:1000; anti-cytochrome (#4212), 1:1000; anti-caspase 3 (#7190), 1:1000; anti-caspase 9 (#9501), 1:1000; anti-PARP (#9542), 1:500 (each Cell Signaling Technology Inc., Danvers, MA, USA); anti-IDO antibody 1:500 (BioGenes, Berlin, Germany); anti-ASK1 (Sc-7931), 1:500; anti-p-ASK1 (Sc-109911), 1:1000; anti-JNK (Sc-474), 1:1000; anti-p-JNK (SC-6254), 1:1000; anti-p38 (Sc-535), 1:1000; anti-p-p38 (Sc-7973), 1:1000; anti-Actin (Sc-1615), 1:5.000; anti-Tom20 (Sc-11415), 1:100; anti-Bap31 (Sc-18579), 1:1000; anti-HO-1 (sc-10789), 1:1000; anti-p-JAK1 (sc-16773), 1:1000, anti-IRE1α (Sc-20790), 1:500; anti-PERK (SC-9477), 1:1000; (Santa Cruz Biotechnology Inc., Santa Cruz, CA, USA); anti-IκBα (Sc-7182), 1:1000; anti-p-IκBα (AF4809, R&D system), 1:1000; anti-JAK1 antibody (ab47435), 1:1000; anti-IRF1 antibody (ab55330), 1:1000 (each ABCAM).

### Extraction of nuclear proteins

The extraction of the nuclear proteins from IFNγ-treated CLS-354 and RPMI 2650, and control cells was performed as described previously [30]. Briefly, following the washing twice with ice-cold PBS buffer the cells were harvested from culture dish with 500 μl of buffer A (20 mM Hepes, pH 7.9; 10 mM NaCl, 0.2 mM EDTA; and 2 mM DTT) supplemented with a recommended concentration of protease inhibitors. After the incubation on ice for 10 min the cells were centrifuged at 14,000×*g* for 3 min to sediment the cell nuclei. The supernatant the contains the cytoplasmic protein was kept at −20 °C for further analysis, while the nuclear pellet was used to extract the nuclear proteins. Accordingly the collected nuclear pellet was resuspended in 50 µl of buffer C (20 mM Hepes, pH 7.9; 420 mM NaCl, 0.2 mM EDTA; 2 mM DTT; 1 mM Na_3_VO_4_, 25 % glycerol) with appropriate amount of protease inhibitors. After the incubation on ice for 20 min the nuclear proteins were purified by the at 14,000×*g* for 3 min. The supernatant that contains the nuclear protein was collected for direct analysis or stored at −80 °C until use.

### Electrophoretic mobility shift assay

The DNA-binding activity of the transcription factors have been analysed as described previously [30]. Briefly, the double stranded synthetic oligonucleotides that represent the specific binding sites of the corresponding transcription factors including, AP-1, ATF-2, p53, NF-κB, STAT1, IRF-1 each purchased from Santa Cruz Biotechnology Inc., Santa Cruz, CA, USA. The double stranded DNA consensus sequence consensus were end-labelled with [γ-32P] ATP (Hartmann Analytika, Munich, Germany) using T4 polynucleotide kinase (Genecraft, Lüdinghausen, Germany). While the measurement of the DNA-binding activity of each transcription factors was performed by the incubation of 4 µg of nuclear extracts with a labelled probe of the transcription factors of interest in a total reaction volume of 30 µl containing EMSA binding buffer (10 mM Tris, pH 7.5; 50 mM NaCl, 1 mM EDTA; 1 mM MgCl_2_; 0.5 mM DTT and 4 % glycerol). After the incubation for 30 min at room temperature the DNA-binding activity of the transcription factors were analyzed by electrophoresis for 3 h at 100 V in 0.5× Tris–borate-EDTA running buffer at room temperature. The dried gel was visualized by exposure to high performance autoradiography film.

### Flow cytometry analysis of apoptosis using annexin V/PI

The analysis of apoptosis of IFNγ-treated and control cells was performed following the staining with 5 µl of annexin V-FITC (Vybrant; Invitrogen, Karlsruhe, Germany) and 5 µl propodeum iodide (100 µg/ml). After the incubation for 15 min at room temperature the number of apoptotic cells were assessed by flow cytometry described previously [28].

### Measurement of mitochondrial membrane potential (ΔΨm) using JC-1

IFNγ- treated CLS-354 and RPMI 2650 cells were stained with 10 μM JC-1 (10 mM; Biotrend, Cologne, Germany) for 30 min at room temperature in the dark. The intensities of green (520–530 nm) and red fluorescence (>550 nm) of 50,000 individual cells were analyzed by flow cytometry as described previously [28].

### Measurement of reactive of oxygen species

The measurement of reactive oxygen species (ROS) in IFNγ- treated and control cells was performed by flow cytometry following the staining with DHR 123 (Sigma) as described [30].

### Immunofluorescence staining

IFNγ- treated and control cells were subjected to immunofluorescence staining as described [31]. Primary antibodies, anti-Noxa (SC-2697), 1:200; anti-Tom20 (Sc-11415), 1:200; anti-Bap31 (Sc-18579), 1:200 (each Santa Cruz Biotechnology Inc., CA, USA) were incubated treated and control cells for 2 h at room temperature. After three successive washing with PBS, the cells were incubated with Alexa Flour labelled secondary antibodies for 2 h at room temperature protected from light. To remove nonspecific binding of the secondary antibodies, the cells were washed three times with PBS, and subsequently mounted using DAKO mounting medium. Photomicrographs were taken on a fluorescence microscope (Leica, Wetzlar, Germany).

### Preparation of mitochondrial and endoplasmic reticulum fractions

The preparation of mitochondrial and endoplasmic reticulum (ER) fractions was performed as described previously [30]. Briefly, IFNγ- treated and control cells (CLS-354 and RPMI 2650) were scraped off with 5 ml of phosphate-buffered saline and collected by the centrifugation at 600×*g* for 5 min. After three washing in PBS buffer the cells have been washed, resuspended and homogenized in PBS buffer. After the centrifugation at 600×*g* for 5 min, the cell the supernatant was layered over a discontinuous gradient of 40 and 60 % sucrose in HE buffer (3 and 1 ml, respectively). Following the centrifugation at 100,000×*g* for 3 h, aliquots of the corresponding of the mitochondrial or ER fractions were precipitated with 10 % trichloroacetic acid (TCA) were directly subjected to western blot analysis or stored at −80 °C until use.

### Statistical analysis

The statistical analysis was performed by considering the average of a at least three independent experiments (n = 3) and average values are expressed as the mean ± SD. The data analysis was performed using Student’s t test method.

## Results

### Effect of IFNγ on the viability of HNSCC cells

**Fig. 1:**
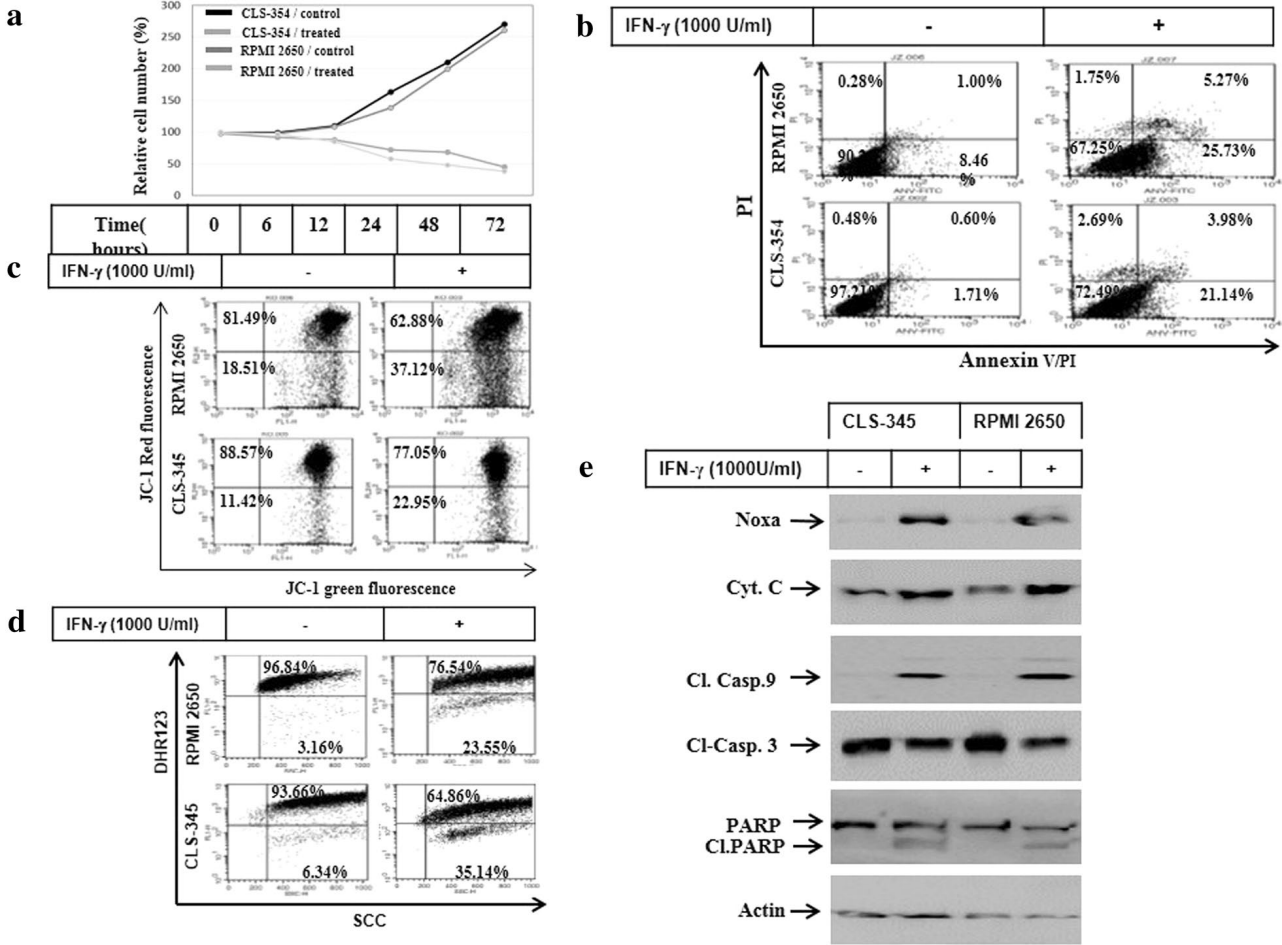
**a** Time course experiment demonstrates cell growth inhibition of HNSCC cell lines CLS-354 and RPMI 2650. Relative cell number (%) assessed by MTT assay following the exposure of cells to IFNγ (1000 U/ml) for regulated time intervals up to 72 h. The values are expressed as the mean ± SD of three independent experiments performed in duplicate. The Student’s t test was used for analysis. **b** Flow cytometry analysis of the induced apoptosis of CLS-354 and RPMI 2650 cells using annexin V/PI staining following the exposure to IFNγ for 48 h. **c** Flow cytometry analysis of the mitochondrial membrane potential (Δ*ψ*m) in CLS-354 and RPMI 2650 cells using JC-1 staining following the exposure to IFNγ for 48 h. **d** Assessment of ROS level in CLS-354 and RPMI 2650 cells following the exposure to IFNγ for 48 h. ROS generation was measured by flow cytometry using dihydrorhodamine (DHR 123). **e** Western blot analysis of the pro-apoptotic protein Noxa, cytochrome c, cleavage of caspase-9, caspase-3 and PARP following the exposure of CLS-354 and RPMI 2650 cells to IFNγ for 48 h. Actin was used as internal control for loading and transfer. Data are representative of three dependent experiments performed separately

Initially, we performed a time-course experiment to assess the cytotoxicity of IFNγ by treating HNSCC cells with IFNγ for time periods up to 72 h. We first assessed IFNγ-induced effects on HNSCC cell viability using a MTT assay. Both CLS-354 and RPMI 2650 cells were cultured for 24 h before the exposure to IFNγ (1000 U/ml). MTT assay (Fig. 1a) indicated that IFNγ treatment resulted in an inhibition of proliferation of both CLS-354 and RPMI 2650 cells. This inhibition was apparent at 12 h after treatment and reached a maximum by 72 h in both cell lines. Next, we investigated whether treatment with IFNγ induced apoptosis. Following treatment with IFNγ for 48 h, the cells were analyzed for apoptosis by flow cytometry using annexin V/PI staining. As expected, IFNγ treatment induced apoptosis in both cell lines (Fig. 1b), suggesting the involvement of an apoptotic mechanism in the modulation of IFNγ-induced death of HNSCC cells. Next, we confirmed whether IFNγ-induced apoptosis is associated with the loss of mitochondrial membrane potential (Δ*ψ*m). Accordingly, we analysed Δ*ψ*m of control and treated cells by flow cytometry following staining with JC-1. Flow cytometric analyses (Fig. 1c) demonstrated the loss of Δ*ψ*m markedly in response to the exposure HNSCC cells to IFNγ, suggesting an important role for mitochondrial dysregulation in the modulation of IFNγ-induced apoptosis. We hypothesized that IFNγ-induced loss of Δ*ψ*m is associated with the accumulation of reactive oxygen species (ROS). The cells were subjected to flow cytometry analysis ROS using dihydrorhodamine 123 (DHR123) after the exposure to IFNγ for the recommended time periods. Flow cytometry data (Fig. 1d) revealed the elevation of ROS accumulation in response to the treatment of HNSCC cells with IFNγ, which suggests a central role for IFNγ-induced ROS accumulation in the modulation of IFNγ-induced apoptosis of HNSCC cells. Next, we analysed the expression of molecular markers of apoptosis, including cytochrome c, caspase-9, caspase-3, and PARP in HNSCC cells. In addition, we analysed the expression of the pro-apoptotic protein Noxa, the expression of which is mostly associated with mitochondrial damage [31]. The analysis of the total protein of treated and control cells by Western blot (Fig. 1e) demonstrated the induction of cytochrome c release, as well as the cleavage of caspases-9, and -3, and PARP. The pro-apoptotic protein Noxa was also expressed by HNSCC after exposure to IFNγ.

### IFNγ-induced expression of IDO is associated with the suppression of heme oxygenase-1

**Fig. 2:**
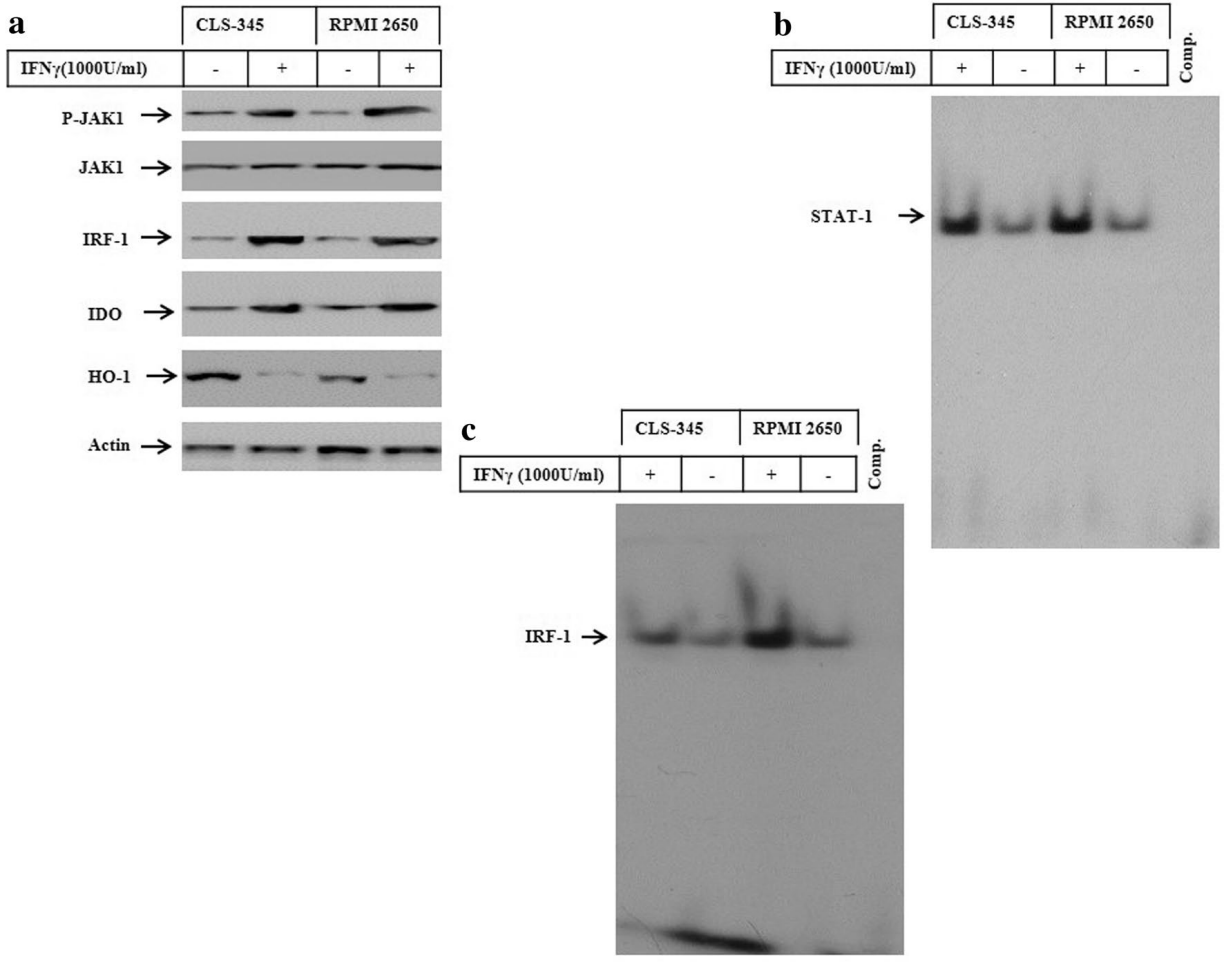
**a** Western blot analysis of JAK1, IRF-1, IDO and HO-1 in CLS-354 and RPMI 2650 cells following the exposure to IFNγ for 48 h. Actin was used as an internal control for loading and transfer. EMSA demonstrates the enhancement of the DNA-binding activity of the transcription factors IRF-1 (**b**) and STAT1 (**c**) following the exposure of CLS-354 and RPMI 2650 cells to IFNγ for 48 h. Data are representative of three independent experiments with similar results

As widely reported, the mutual crosstalk between heme oxygenase-1 (HO-1) and IDO can influence cellular factors that are thought to be associated with cell death and proliferation [32]. We investigated whether IFNγ-induced IDO expression influences basal heme oxygenase-1(HO-1) expression. The cells were treated with of IFNγ for 48 h the cells and total cell lysates were used for the protein expression analysis of IDO, interferon regulatory factor-1 (IRF-1), and HO-1 by western blot. In addition, we analysed the expression and phosphorylation of the Janus kinase 1 (Jak1) as well as IDO activity. Western blot analyses (Fig. 2a) demonstrated the ability of IFNγ to induce expression of both IDO and IRF-1 as well as both expression and phosphorylation of Jak1, while suppressing HO-1 expression. In addition, we assessed the activity of the transcription factors STAT1, NF-κB, and IRF-1, which are thought to be responsible for the induction of IDO expression in response to the treatment of HNSCC cells by IFNγ. The analysis of the transcription factors were performed by electrophoretic mobility shift assay (EMSA) using nuclear extracts prepared from CLS-354 and RPMI 2650 cells before and after the exposure to IFNγ. EMSA (Fig. 2b, c) demonstrated the activation of the transcription factors STAT1, NF-κB, and IRF-1 in response to the treatment with IFNγ, suggesting an important role for these transcription factors in the modulation of IFNγ- induced IDO expression and its consequences in HNSCC cells.

### The exposure of HNSCC cells to IFNγ is associated with the activation of ASK1-dependent pathways

**Fig. 3:**
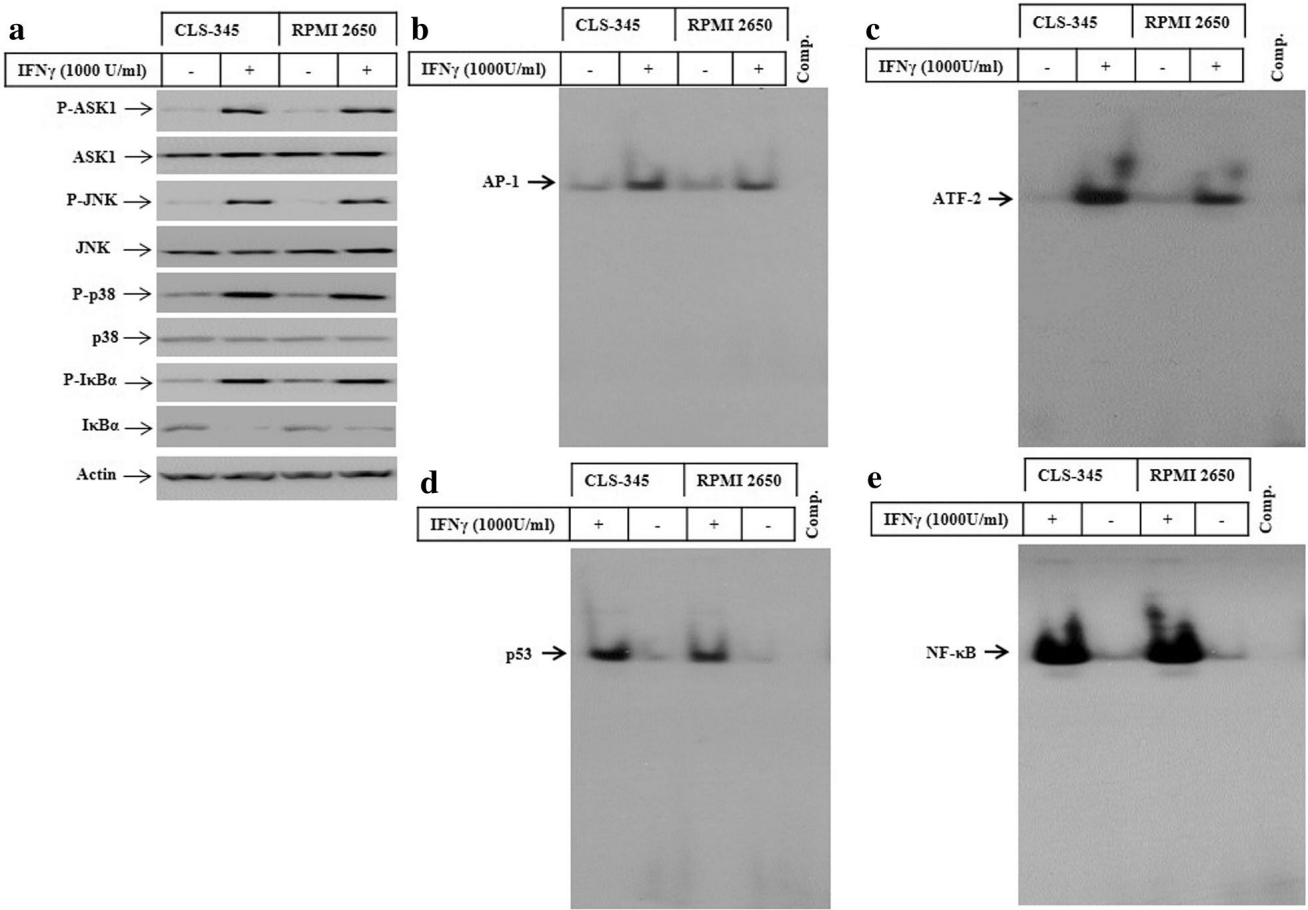
**a** Western blot analysis of ASK1, JNK, p38 and IκBα in CLS-354 and RPMI 2650 cells following the exposure to IFNγ for 48 h. Actin was used as an internal control for loading and transfer. EMSA demonstrates the enhancement of DNA-binding activity of the transcription factors AP-1 (**b**), ATF-2 (**c**), p53 (**d**) and NF-κB (**e**) following the exposure of CLS-354 and RPMI 2650 cells to IFNγ for 48 h. Data are representative of three independent experiments with similar results

Accumulation of ROS is associated with the activation of apoptosis signalling regulating kinase 1 (ASK1) and its downstream signalling pathways such as c-Jun-N-terminal kinase (JNK), p38 and NF-κB [28, 33]. To analyse key components of ASK1-dependent pathways, CLS-354 and RPMI 2650 cells were treated with IFNγ for 48 h, then total cell lysates were prepared for western blot analyses of ASK1, JNK, p38, and IκBα. The exposure of CLS-354 and RPMI 2650 cells to IFNγ induced the phosphorylation of ASK1, JNK, p38 and IκBα proteins (Fig. 3a). More importantly, the phosphorylation of IκBα was associated with its subsequent degradation, while there was no alteration in the basal expression level of ASK1, JNK, or p38 proteins (Fig. 3a). Also, we investigated whether IFNγ-induced activation of ASK1-JNK, ASK1-p38 or NF-κB pathways is associated with the enhancement of the DNA-binding activity of the transcription factors AP-1, p53, ATF-2 and NF-κB. The analysis of the DNA-binding activities of the transcription factors of interest by EMSA using the nuclear extracts of IFNγ-treated cells demonstrated the activation of the transcription factors AP-1 (Fig. 3b), ATF-2 (Fig. 3c), p53 (Fig. 3d) and NF-κB (Fig. 3e), suggesting an important role for ASK1-dependent pathways in the modulation of IFNγ-induced activation of the transcription factors AP-1, ATF-2, p53 and NF-κB.

### IFNγ-induced accumulation of ROS is attributed to the suppression of HO-1 by IDO

**Fig. 4:**
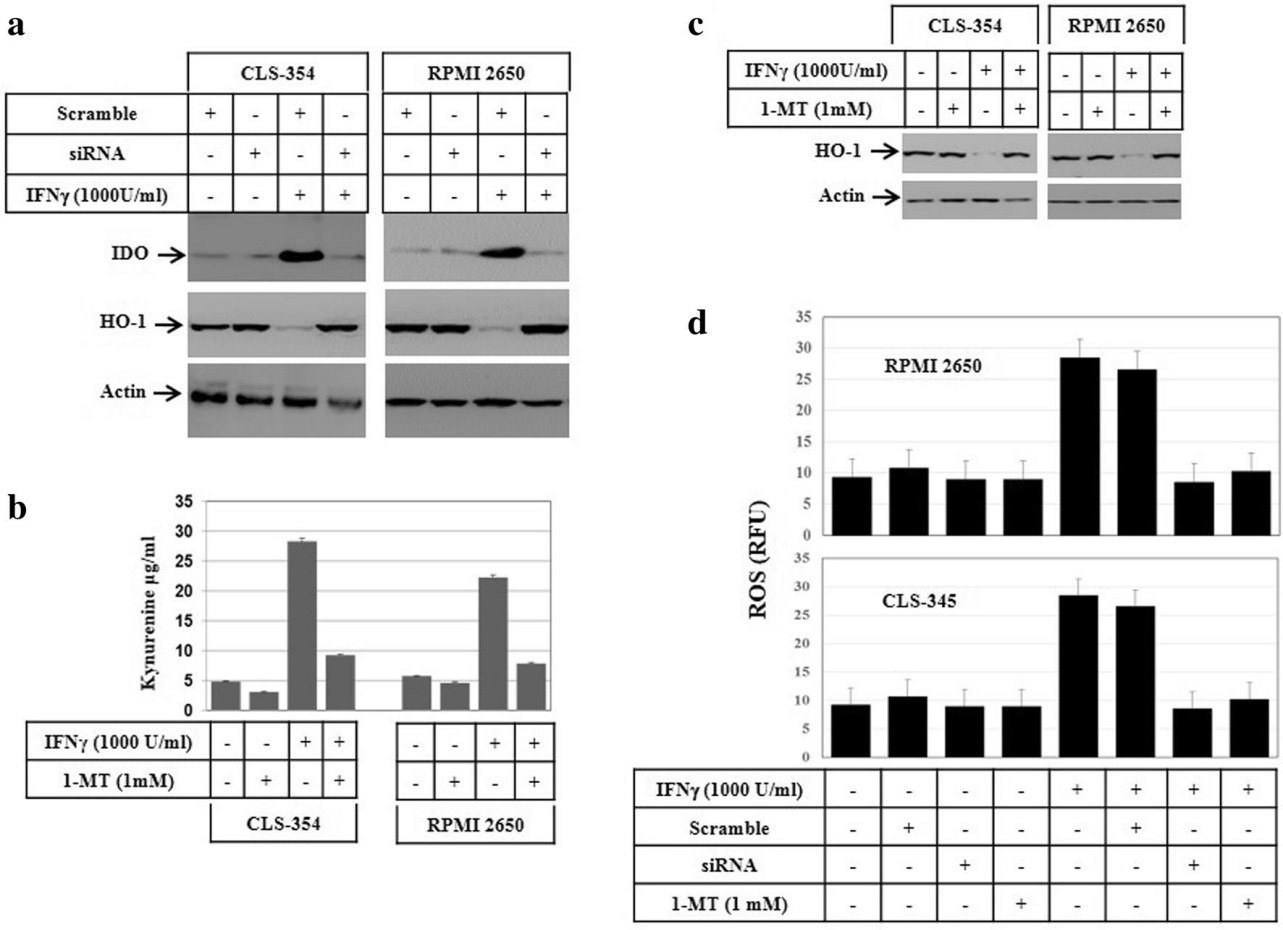
**a** Western blot analysis of IDO and HO-1 proteins following the knockdown of IDO expression by its specific siRNA before and after the exposure of CLS-354 and RPMI 2650 cells to IFNγ for 48 h. Actin was used as internal control for loading and transfer. **b** Spectrofluorometric analysis shows the ability of 1-methyl-d-tryptophan (1-MT) to block IFNγ-induced IDO activity in both CLS-354 and RPMI 2650 cells. Cells were grown with medium alone or medium containing IFNγ in the absence or presence of 1-MT for 48 h. The supernatant was collected, and residual tryptophan was converted to norharman. Norharman levels were measured by a spectrofluorometer using 360 and 640 nm as excitation and emission wavelengths, respectively. The values are expressed as the mean ± SD of three independent experiments performed in duplicate. The student’s t-test was used for analysis. **c** Western blot analysis demonstrates the inhibition of INFγ-induced activation of IDO results in the rescue of IFNγ-induced suppression of HO-1 protein. Actin was used as internal control for loading and transfer. **d** Flow cytometry analysis of IFNγ-induced accumulation of the reactive oxygen species (ROS) using DHR 123 in RPMI 2650 and CLS-354 cells following the knockdown of IDO by its specific siRNA or the inhibition of IDO enzyme activity by 1-MT. ROS generation was measured by flow cytometry using dihydrorhodamine (DHR 123). The values are expressed as the mean ± SD of three independent experiments performed in duplicate. The Student’s t test was used for analysis

In order to determine if IFNγ-induced accumulation of ROS is the consequence of HO-1/IDO cross-regulation, we investigated if the suppression of IDO expression using siRNA might influence IFNγ-induced accumulation of ROS and subsequently alter the expression level of HO-1. The role of HO-1 in the modulation of redox imbalance has been reported in several studies [34, 35]. Also, the crosstalk-regulation between HO-1/IDO is essential for ROS-induced effects. After treatment for 48 h, treated and control cells were harvested for the preparation of total cell lysates for western blot analysis of IDO, interferon regulatory factor-1 (IRF-1) and HO-1 proteins, as well as for the assessment of ROS accumulation by flow cytometry. When cells were transfected with an IDO-specific siRNA, the IFNγ-induced expression of IDO was knocked down when compared to the expression level of IDO in treated and control cells (Fig. 4a). Interestingly, IFNγ-induced expression of IDO expression correlated with the suppression of HO-1 when compared to control cells (Fig. 4a), suggesting an essential role for IFNγ-induced IDO in the suppression of HO-1. To test this, we examined the inhibitory effect of the 1-methyltryptophan (1-MT), the inhibitor of IDO enzyme, on IFNγ-induced IDO activity and its affect on HO-1. Following the pre-treatment with 1-MT, CLS-354 and RPMI 2650 cells were challenged with IFNγ for 48 h, then the IDO activity was determined in the total cell lysates by ELISA. This assay demonstrated the suppression of IFNγ- induced IDO activity in both HNSCC cells by 1-MT (Fig. 4b). Also, the inhibition of IFNγ-induced IDO activity is involved in the suppression of HO-1 (Fig. 4c). To determine whether the inhibition of IDO-induced suppression of HO-1 is essential for modulation of IFNγ-induced ROS accumulation in HNSCC cells, prior to the exposure to IFNγ the cells were either transfected with IDO specific siRNA or pre-treated with 1-MT. The analysis of ROS accumulation by flow cytometry using DHR123 in RPMI 2650 and CLS-354 cells (Fig. 4d) demonstrated the reduction of IFNγ-induced ROS accumulation by the suppression of IDO expression or activation by its siRNA or 1-MT, respectively, when compared to control cells. These findings indicate that the increased accumulation of ROS is the consequence of IFNγ-induced IDO leading to the suppression of HO-1.

### The pre-treatment of HNSCC cells with NAC blocks IFNγ-induced activation of ASK1-JNK, ASK-p38 and NF-κB pathways

**Fig. 5:**
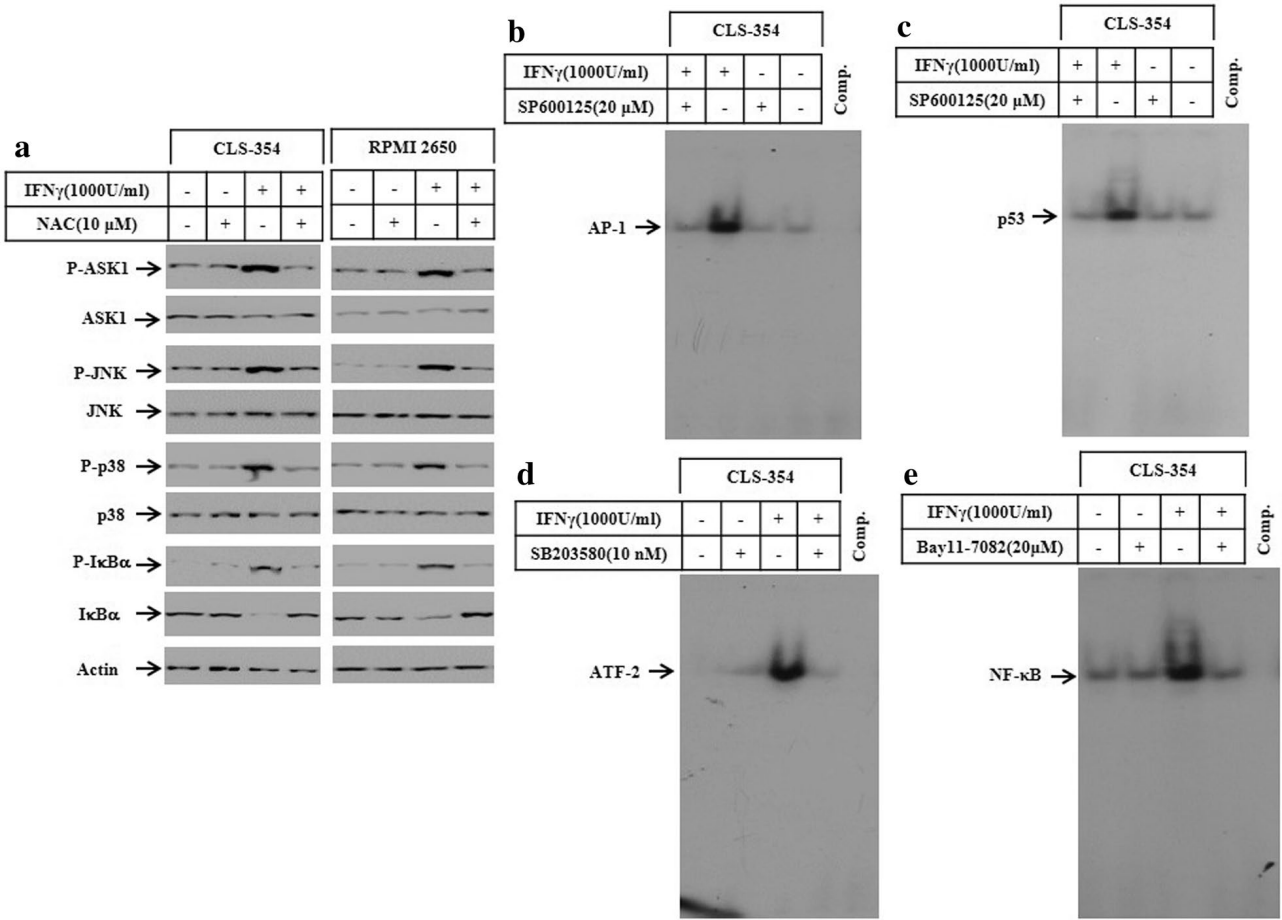
**a** Western blot analysis demonstrates the inhibition of IFNγ-induced phosphorylation of ASK1, JNK and p38 as well as the IFNγ-induced phosphorylation and degradation of IκBα in response to the pre-treatment of CLS-354 and RPMI cells with the scavenger of ROS. Actin was used as internal control for loading and transfer. EMSA analysis demonstrates the inhibition of IFNγ-induced DNA-binding activity of the transcription factors AP-1 (**b**) and p53 (**c**) in response to the pre-treatment of CLS-354 cells by the inhibitor of JNK pathway (SP60015). Whereas, the pre-treatment of the same cells with the inhibitors of p38 (SB203580) or the inhibitors of NF-κB pathway (Bay11-7082) was found to block IFNγ-induced DNA-binding activity of ATF-2 (**d**) and NF-κB (**e**), respectively. Data are representative of three independent experiments performed separately

In order to further analyze the role of ROS in IFNγ-induced activation of ASK1 and its downstream pathways, CLS-354 and RPMI 2650 cells were pre-treated with *N*-acetyl-l-cysteine (NAC) before exposure to IFNγ for 48 h. Subsequently, total cell lysates and nuclear extracts were prepared to perform western blot analyses and EMSA assays, respectively. The suppression of ROS accumulation by NAC inhibited IFNγ-induced phosphorylation of ASK1, JNK, p38, and of IκBα together with the subsequent degradation of IκBα (Fig. 5a), suggesting an important role for IFNγ-induced ROS accumulation in the activation of ASK1-JNK/- p38, and NF-κB pathways. In addition, the DNA-binding activity of the transcription factors AP-1, p53, ATF-2 and NF-κB were analyzed by EMSA. The inhibition of IFNγ-induced activation of the transcription factors AP-1 (Fig. 5b) and p53 (Fig. 5c) by the inhibitor of JNK pathway (SP600125) suggested an important role for JNK in their regulation. Similar results have been demonstrated in RPMI 2650 (data not shown). The inhibitor of p38 (SB203580) was found to inhibit IFNγ-induced activation of the transcription factor ATF-2 in CLS-354 cells (Fig. 5d), suggesting an important role for p38 in the regulation of ATF-2 activation. A similar effect was noted in RPMI 2650 cells in response to the pre-treatment with inhibitor of p38 (data not shown). Also, the inhibition of NF-κB pathway by its specific inhibitor (Bay11-7082) inhibited IFNγ-induced DNA-binding activity of NF-κB in CLS-354 (Fig. 5e). The same effects were noted in RPMI 2650 (data not shown).

### IFNγ-induced apoptosis of HNSCC cells is the consequence of Noxa-mediated mitochondrial dysregulation

**Fig. 6:**
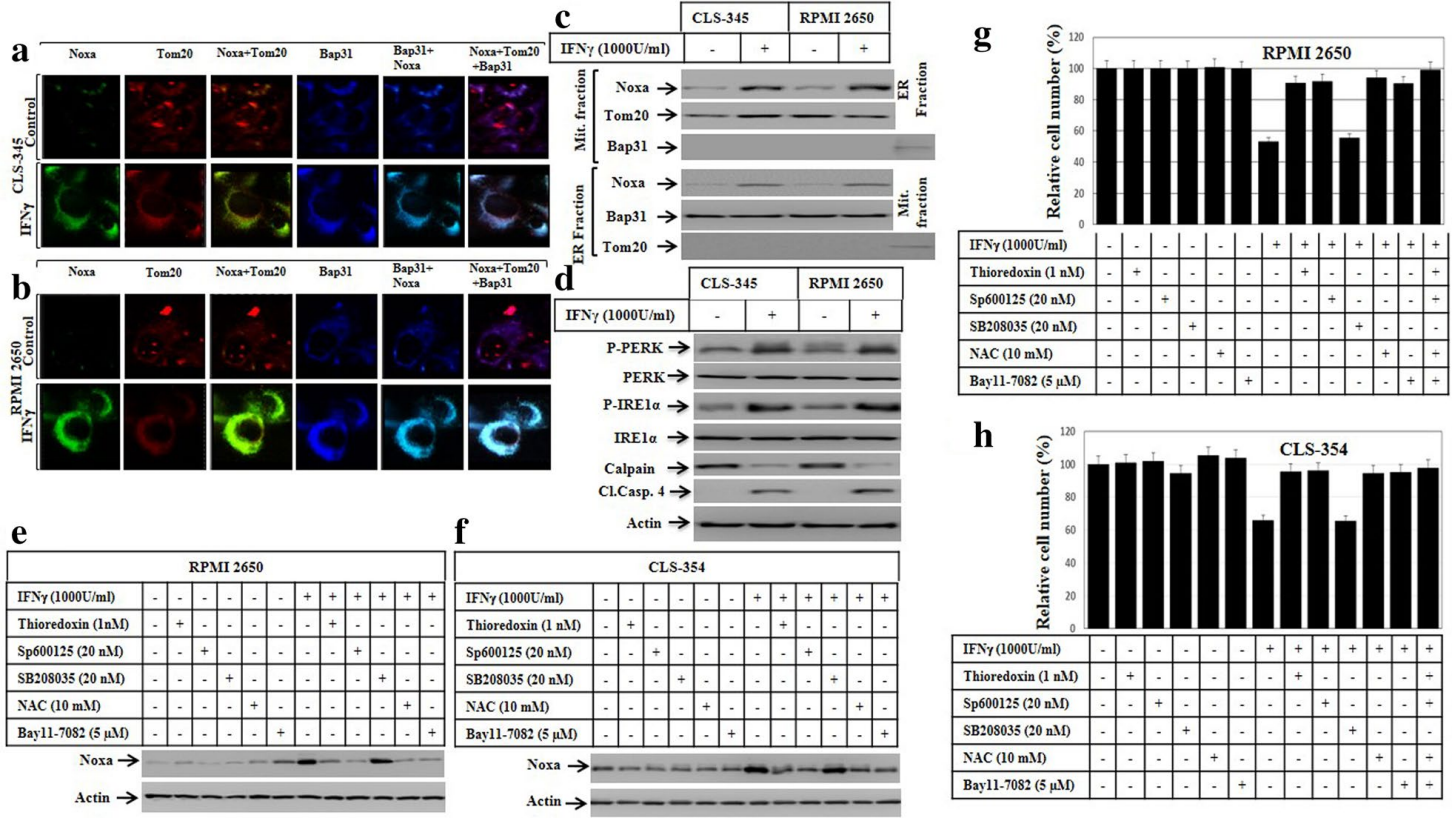
**a** Subcellular localization of Noxa protein to both mitochondria and endoplasmic reticulum (ER). Immune fluorescence: CLS-354 and RPMI 2650 cells were treated with IFNγ for 48 h before the staining with anti- Noxa, Tom20 (mitochondrial marker) and Bap31 (ER marker). The subcellular localization of Noxa (*green*) to mitochondria (*red*) and the overlay of Noxa with Tom20 staining demonstrates the localization of Noxa to mitochondria (*yellow*), when compared to control cells. **b** The subcellular localization of Noxa (*green*) to ER (*blue*) and the overlay of Noxa with Bap31 staining demonstrates the localization of Noxa to ER (*turquoise*), when compared to control cells. **c** Western blot analysis using mitochondrial fraction (Mit. fraction) and ER fraction from both CLS-354 and RPMI 2650 cells following the treatment with IFNγ for the indicated time periods. The detection of Noxa in mitochondrial and ER fractions of CLS-354 and RPMI 2650 cells after the exposure to IFNγ confirm the localization of Noxa protein to both mitochondria and ER. The purity of both mitochondrial and ER fractions was verified by the detection of the mitochondrial protein Tom20 in the mitochondrial fraction and the detection of Bap31 in ER fraction. **d** Western blot analysis demonstrates the phosphorylation of both PERK and IRE1α, calpain degradation, and cleavage of caspase-4 in response to the treatment of HNSCC cells with IFNγ. **e** Western blot analysis demonstrates the inhibition of IFNγ-induced expression of Noxa in RPMI 2650 in response to the pre-treatment with the inhibitors of the ASK1 (thioredoxin), JNK (SP600125), ROS (NAC) and NF-κB (Bay11-7082), but not with those of p38 (SB208035). **f** Western blot analysis demonstrates the inhibition of IFNγ-induced expression of Noxa in CLS-354 cells in response to the pre-treatment with the inhibitors of the ASK1 (thioredoxin), JNK (SP600125), ROS (NAC) and NF-κB (Bay11-7082), but not with those of p38 (SB208035). Actin was used as internal control for loading and transfer. Data are representative of three independent experiments. **g** MTT demonstrates the inhibition of IFNγ-induced death of RPMI 2650 cells in response the pre-treatment with the inhibitors of the ASK1 (thioredoxin), JNK (SP600125), ROS (NAC) and NF-κB (Bay11-7082), but not with those of p38 (SB208035). **h** MTT demonstrates the inhibition of IFNγ-induced death of CLS-354 cells in response to the pre-treatment with the inhibitors of the ASK1 (thioredoxin), JNK (SP600125), ROS (NAC) and NF-κB (Bay11-7082), but not with those of p38 (SB208035). The values are expressed as the mean ± SD of three independent experiments performed in duplicate. The Student’s t test was used for analysis

To show whether IFNγ-induced apoptosis is the consequence of the subcellular localization of Noxa protein to the mitochondria and/or ER, both CLS-354 and RPMI 2650 cells were treated with IFNγ for the indicated time periods before analysis of the subcellular localization of Noxa protein using immunofluorescence and western blot. Following the treatment with IFNγ for 48 h, the cells were fixed and subsequently subjected to the staining with antibodies against Noxa, the mitochondrial marker Tom20, and the ER marker Bap31. Immunofluorescence staining confirmed the localization of Noxa protein to both mitochondria and ER in CLS-354 (Fig. 6a) and RPMI 2650 (Fig. 6b) cells. In addition to immunofluorescence staining we prepared mitochondrial and ER subcellular fractions for western blot analyses for confirmation of the immunofluorescence data. Immunoblot analysis of both mitochondrial and ER fractions prepared from treated and control cells revealed the presence of Noxa protein only the mitochondrial and ER fractions of IFNγ in treated cells (Fig. 6c). Tom20 and Bap31 proteins were used as marker for the purity of mitochondrial and ER fractions, respectively. In conclusion, these data suggest that IFNγ-induced IDO is essential for the modulation of mitochondrial dyseregulation as well as ER stress via a mechanism mediated by the subcellular localization of Noxa protein to both mitochondria and ER (Fig. 6c). To further characterize the ER dysfunction, we analysed ER stress associated proteins such as protein kinase RNA-like endoplasmic reticulum kinase (PERK), inositol-requiring-1α (IRE1α), calpain, and caspase-4. The cell lines were treated with the recommended concentration of IFNγ for 48 h and the total cell lysates of treated- and control cells were subjected to western blot analysis. Although no changes was noted on the basal expression of either PERK or IRE1α, the exposure of the cells to IFNγ is able to enhance the phosphorylation of PERK as well as IRE1α (Fig. 6d) suggesting the induction of ER stress as consequence of Noxa localization to the ER. Next, we set out to address the pathways which are responsible for the regulation of IFNγ-induced Noxa expression. Accordingly, we analysed the effect of ASK1, JNK, p38, ROS and NF-κB inhibitors on IFNγ-induced Noxa expression. The pre-treatment of both CLS-354 (Fig. 6e) and RPMI 2650 (Fig. 6f) cells with the inhibitors of ASK1, JNK, ROS and NF-κB, but not with those of p38, are able to block IFNγ-induced expression of Noxa, suggesting an essential role for ASK1-JNK and ASK1- NF-κB pathways, but not ASK1-p38 pathway in the regulation of IFNγ-induced Noxa expression. Moreover, we examined the effect of the inhibition of ASK1-JNK, ASK1-p38 and ASK1-NF-κB pathways on IFNγ-induced cell death. The CLS-354 and RPMI 2650 cells were pre-treated with the inhibitors of ASK1, JNK, p38, ROS and NF-κB before the exposure to IFNγ for 48 h, then the cell viability was analysed by MTT assay (Fig. 6g, h), the inhibition of IFNγ-induced ROS accumulation or activation of ASK1, JNK, and NF-κB pathways, but not the inhibition of p38 pathway can block IFNγ-induced death of CLS-354 and RPMI 2650 cells, suggesting an important role for the pathways ASK1-JNK, and NF-κB in the regulation of IFNγ-induced apoptosis of CLS-354 and RPMI 2650 cells via Noxa-dependent mitochondrial damage.

## Discussion

The present study provides a new insight into the mechanistic role of IFNγ-induced apoptosis of HNSCC cells and describes the possible role of IDO in the modulation of IFNγ-induced apoptosis during the course of immune therapy. The treatment of the HNSCC with IFNγ at a concentration of 1000 IU/ml is based on the determined IC50 as well as on the recommended dose by Yonekura et al. as described [36]. IFNγ-induced apoptosis of HNSCC cells is a result of the IDO-induced suppression of HO-1, which leads to the increased accumulation of ROS that, in turn, triggers the induction of oxidative stress-associated pathways. These pathways include ASK1-JNK, ASK-p38 and ASK1-IKK/NF-κB that are essential for the induction of the pro-apoptotic protein Noxa. The subcellular localization of Noxa protein triggers mitochondrial dysregulation, an essential step for the initiation of apoptosis. Accordingly, the present study demonstrated the involvement of ASK1-JNK-p53/AP-1 and ASK1-IKK-NF-κB pathways in the modulation of IFNγ-induced Noxa expression. The activation of these signalling pathways is expected to be the consequence of IDO-induced suppression of HO-1 leading to ROS. Although the subcellular localization of Noxa protein to both ER and mitochondria has been observed, Noxa-induced mitochondrial damage seems to be essential for IFNγ-induced apoptosis of HNSCC cells. The localization of Noxa protein to mitochondria is associated with the loss of ΔΨm and the subsequent release of cytochrome c, and, cleavage of caspases-9, 3 and PARP, whereas IFNγ-induced Noxa to ER seems to be associated with the induction of ER stress, as evidenced by the phosphorylation of PERK and IRE1α.

IFNγ exerts its pleiotropic effects on normal and malignant cells via the interaction with a specific receptor that is commonly expressed on the surface of most eukaryotic cells [37–39]. The role of IFNγ in the regulation of IDO has been reported in several studies [40, 41]. IFNγ- induced IDO is mediated by JAK-STAT pathway-dependent mechanism(s) [42]. IDO belongs to a pattern of gene transcripts such as JAK2, IRF-1 and STAT1α [42]. Thus, the activation of the JAK-STAT pathway is associated mainly with the increased phosphorylation of JAK1 and the induction of both expression and DNA-binding activity of IRF-1.

The rescue of IFNγ-induced apoptosis by IDO inhibition provides evidence for the involvement of IDO in the modulation of IFNγ-induced apoptosis. Although the role of IDO as apoptotic mediator has been described previously [18, 43], we describe for the first time a central role for IDO in the modulation of IFNγ-induced apoptosis of HNSCC cells. Apoptosis of HNSCC cells by IFNγ is regulated by IDO-mediated suppression of HO-1 leading to the accumulation of ROS. As a consequence, the accumulation of ROS triggers the activation of ASK1-JNK and ASK1-NF-κB pathways [18, 33]. The role of HO-1 in the inhibition of apoptosis via mechanism mediated by the suppression of oxidative stress-associated pathways has been reported in several studies [44–46]. In accordance, we found that the induction of IDO expression or activation results in the suppression of HO-1 protein that, in turn leads to ROS accumulation. The involvement of IDO in the modulation of IFNγ-induced apoptosis is supported by the findings that demonstrate the rescue of IFNγ-induced suppression of HO-1 along with the inhibition of IFNγ-induced apoptosis in response to the knockdown of IDO expression or inhibition of IDO activity.

The role of mitochondrial dysregulation associated pathways in the modulation of IFNγ-induced apoptosis has been reported in several studies [47–49]. Similarly, we found that IFNγ-induced mitochondrial damage is the consequence of the activation of ASK1-JNK, ASK1-JNK and NF-κB pathways leading to the activation of the transcription factors AP-1, p53 and NF-κB. These transcription factors are thought to form a transcription complex that is essential for the promoter activation of Noxa gene. Since the induction of apoptosis of HNSCC cells by IFNγ is associated with Noxa-induced loss of Δ*ψ*m.

## Conclusion

**Fig. 7:**
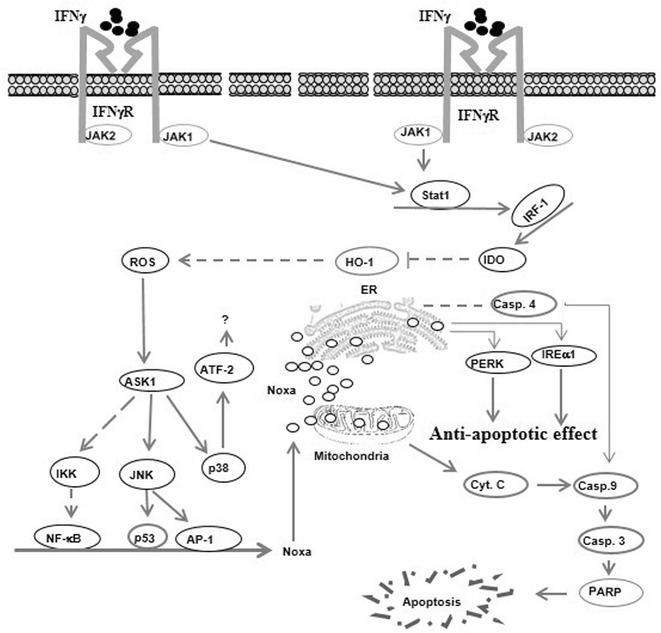
Proposed model for IFNγ-induced apoptosis of HNSCC cells. The activation of IFNγ receptor of HNSCC cells with IFNγ results in the activation of JAK-STAT pathway that subsequently enhances the DNA binding activity of the transcription factor interferon regulatory factor-1 (IRF-1) that is essential for the induction IDO expression. The increased level of IDO results in the suppression of the anti-oxidant protein heme oxygenase-1 (HO-1) that, in turn, leads to the accumulation of reactive oxygen species (ROS). The increased accumulation of ROS mediates oxidative stress-associated pathways such as apoptosis signal-regulating kinase 1(ASK1)/c-Jun-N-terminal kinase (JNK)-AP-1/p53, IKK-NF-κB, and p38-ATF-2. The formation of a transcriptional complex from the transcription factors NF-κB, AP-1 and p53 results in the transcriptional activation of the promoter of Noxa protein. The localization of Noxa protein to mitochondria results in the loss of mitochondrial membrane potential (Δ*ψ*m) that is characterized by the release of cytochrome c (Cyt.C) into the cytoplasm where it initiates apoptosis by formation of apoptosome complex leading to the cleavage of caspase-9, caspase-3 and PARP. Whereas the localization of Noxa to endoplasmic reticulum (ER) results in ER-stress that is associated with the activation of the protein kinase RNA-like endoplasmic reticulum kinase (PERK) and inositol-requiring-1α (IRE1α) pathways as well as cleavage of caspase-4 that, in turn, mediates the cleavage of caspase-3

Our data provide insight into the possible mechanisms of the anti-tumor activity of IFNγ during the course of immune therapy and demonstrate for the first time an essential role for IDO as a mediator of IFNγ-induced apoptosis of HNSCC cells. IFNγ-induced apoptosis of HNSCC cells is regulated via a mechanism mediated by IDO-induced suppression of the antioxidant protein HO-1 that, in turn, leads to an increase of ROS accumulation. The increased accumulation of ROS is essential for the activation of oxidative stress-dependent pathways including ASK1-JNK, ASK1-JNK and NF-κB axis. Thus, based on our findings we proposed a model for IFNγ-induced apoptosis of HNSCC cells (Fig. 6). This model outlines the possible mechanism(s) that are implicated in the regulation of HNSCC response to immunotherapy (Fig. 7).

## Abbreviations

ATF-2: activating transcription factor 2
AP-1: activator protein 1
ASK1: apoptosis signal-regulating kinase
EMSA: electrophoretic mobility shift assay
ER: endoplasmic reticulum
HNSCC: head and neck squamous cell carcinoma
HO-1: heme oxygenase-1
IDO: indoleamine 2,3-dioxygenase
IFNγ: interferon gamma
IKK: IkappaB kinase
IRE1α: protein kinase RNA-like endoplasmic reticulum kinase (PERK), inositol-requiring-1α
IRF: interferon regulatory factor 1
JAK: janus kinase
LPS: lipopolysaccharides
MHC: major histocompatibility complex
NF-κB: nuclear factor kappa-light-chain-enhancer of activated B cells
PARP: poly ADP ribose polymerase
PERK: protein kinase RNA-like endoplasmic reticulum kinase
ROS: reactive oxygen species
STAT: signal transducer and activator of transcription
Δ*ψ*m: mitochondrial membrane potential
